# Impact of Covid-19 on health-related quality of life of patients: A structured review

**DOI:** 10.1371/journal.pone.0259164

**Published:** 2021-10-28

**Authors:** Ak Narayan Poudel, Shihua Zhu, Nicola Cooper, Paul Roderick, Nisreen Alwan, Carolyn Tarrant, Nida Ziauddeen, Guiqing Lily Yao

**Affiliations:** 1 Department of Health Sciences, University of Leicester, Leicester, England, United Kingdom; 2 Primary Care and Population Sciences, University of Southampton, Southampton, England, United Kingdom; Post Graduate Institute of Medical Education and Research, INDIA

## Abstract

**Introduction:**

Coronavirus disease (Covid-19) has led to a global pandemic since its emergence in December 2019. The majority of research into Covid-19 has focused on transmission, and mortality and morbidity associated with the virus. However, less attention has been given to its impact on health-related quality of life (HRQoL) of patients with Covid-19.

**Methods:**

We searched for original studies published between December 2019 and Jan 2021 in PubMed, Scopus and Medline databases using a specific search strategy. We also explored literature on websites of distinguished public health organisations and hand-searched reference lists of eligible studies. The studies were screened by two reviewers according to the Preferred Reporting Items for Systematic Reviews and Meta-Analysis (PRISMA) flowchart using pre-determined eligibility criteria. Data were synthesised, analysed descriptively and reported in line with PRISMA guidelines.

**Results:**

In total, 1276 studies were identified through the search strategy. Of these, 77 studies were selected for full-text reading after screening the studies. After reading full-text, 12 eligible studies were included in this review. The majority of the studies used a generic HRQoL assessment tool; five studies used SF-36, five studies used EQ-5D-5L, and three used pulmonary disease-specific HRQoL tools (two studies used two tools each). The impact of Covid-19 on HRQoL was found to be considerable in both Acute Covid and Long Covid patients. Higher impact on HRQoL was reported in Acute Covid, females, older ages, patients with more severe disease and patients from low-income countries.

**Conclusion:**

The impact of Covid-19 on HRQoL of Acute and Long Covid patients is substantial. There was disproportional impact on patients by gender, age, severity of illness and study country. The long-term impact of Covid-19 is still in its initial stage. The findings of the review may be useful to researchers, policymakers, and clinicians caring for people following Covid-19 infection.

## Introduction

Coronavirus disease (Covid-19) is a contagious disease caused by a newly-discovered virus known as SAR-CoV-2 [1]. The World Health Organisation (WHO) declared Covid-19 a pandemic on 11 March 2020 [2]. Leading health organisations including WHO are working with medical experts, government bodies and public health scientists to expand scientific knowledge for tracking the spread and consequences of the virus with an aim of providing timely advice in controlling and minimising the transmission and impact of the virus [3]. The WHO has published a number of guidelines, strategies and action plans. According to the United Nations (UN) [4], the coronavirus pandemic has revealed unambiguous global inequities, fragilities, and unsustainable practices, and has exerted its impact all over the world [4].

It is well-established that Covid-19 causes a wide variety of symptoms [5]. It may cause prolonged illness and persistent symptoms not only in the elderly and individuals with underlying conditions, but also in young adults and people with no or few chronic underlying medical conditions [6]. Coronavirus causes interstitial pneumonia and respiratory distress syndrome, which may lead to multiple organ failure [7]. The virus may affect different organs and body systems such as heart (damage to heart muscle, heart failure), lungs (damage to lung tissue and restrictive lung failure), brain and nervous system (anosmia, consequences of thrombo-embolic events, such as stroke, cognitive impairment), mental health (anxiety, depression, sleep disturbance) and musculoskeletal problems and fatigue [8]. Patients who recover may continue to be affected with hypoxia, shortness of breath and reduced ability to work [9, 10]. Recent reports suggest that some patients may develop medical complications and 11%-24% of Covid-19 patients may experience long-term symptoms even after three months from the onset of Covid-19 illness [8, 11, 12]. Because of the above reasons, Covid-19 may lead to poorer health-related quality of life (HRQoL) of the patients infected both in short and long term.

Impacts of an illness usually go beyond its clinical outcome such as mortality and morbidity, and encompass subjective measures in terms of HRQoL [13]. HRQoL is a multi-dimensional concept that includes domains related to physical, mental, social and emotional functioning [14]. There are a number of HRQoL measurement tools, some of which are generic and some disease specific. Generic HRQoL tools (e.g. SF-36 (36-item Short-Form Health Survey), SF-6D (Short-Form 6 Dimension) derived from the SF-36, and EQ-5D (EuroQol- 5 Dimension)) are widely used to assess multi-dimensional domains of the health and well-being of different populations [15]. Disease specific quality of life assessment instruments related to pulmonary disease include St. George Respiratory Questionnaire (SGRQ) and Clinical COPD Questionnaire (CCQ) [16, 17], which have been used in HRQoL assessment of Covid-19 patients [10, 18].

Various measures have been taken by different countries in controlling the spread of the virus ranging from city-level quarantine, local lockdown, closing borders to patient-level isolation. Research shows that social distancing measures (e.g. ‘stay-at-home order’), use of masks and closures of restaurants, bars, and entertainment-related businesses considerably reduce the spread of Covid-19 [19, 20]. However, such measures not only affect economy and education, but also affect the physical and mental health, and quality of life of restricted patients [21–24]. Studies from the USA and Bangladesh evidenced that social distancing measures, such as ‘a stay-at-home order’, is associated with greater health risks, financial worry, and loneliness [25, 26].

To our knowledge, no review has been published to date assessing the impact of Covid-19 on the HRQoL of patients with Covid-19 (confirmed or suspected). Moreover, very little is known about the impact of Covid-19 on HRQoL of Acute Covid (≤4 weeks from onset of symptoms) and Long Covid (>4 weeks from onset of symptoms) patients. Therefore, the aims of this review are to fill the knowledge gap by identifying and assessing the studies reporting on the impacts of COVID-19 on HRQoL of patients with Covid-19 (confirmed or suspected) and exploring the risk factors for reduced HRQoL of Covid-19 patients.

## Methods

This is a rapid review and it has been reported according to the Preferred Reporting Items for Systematic Reviews and Meta-Analyses (PRISMA) guidelines [27]. This review addresses the following research questions:

1. To what extent does Covid-19 impact on the HRQoL of patients?
2. What are the long-term impacts of Covid-19 on the HRQoL of patients?
3. Is there any differences in impact of Covid-19 on HRQoL of patients by study country?
4. What are the important factors (e.g. age, gender, severity of illness) affecting HRQoL of Covid-19 patients for short and long term?
5. What are the limitations of the studies conducted to date, and what research is required to assess the full impact of Covid-19 on HRQoL of patients?

### Literature coverage and search strategies

We searched PubMed, Scopus and Medline using a combination of the following search terms (in Title/Abstract): Corona, Covid, SARS-CoV-2, "quality of life". An example of search strategy is presented below (for PubMed).

("Corona"[Title/Abstract] OR "Covid"[Title/Abstract] OR "SARS-CoV-2"[Title/Abstract]) AND ("quality of life"[Title/Abstract])

Additional filters used in the search strategies were English language, and original articles published between December 2019 and 25^th^ Jan 2021. We excluded letters, correspondences, notes, case reports, case series, communications, conference reports, reviews and editorials.

In addition to searching databases, reference lists of eligible studies were reviewed to identify additional papers. Grey literature was identified by searching the following websites: Public Health England (PHE), Public Health Wales (PHW), Health Protection Scotland (HPS), Public Health Scotland (PHS), Department of Health and Social Care (DHSC) (UK), Health Protection Agency (HPA), National Institute for Health and Care Excellence (NICE), Centre for Disease Control and Prevention (CDC), World Health Organisation (WHO), Public Health Europe (*PHE).

### Eligibility criteria

We selected original studies using the eligibility criteria given in Table 1. We also used the PICO (Population, Intervention, Comparison and Outcome) framework. In this review, population (P) will be humans with all ages and sexes, and Covid-19 confirmed or suspected patients who were isolated, intervention (I) is not applicable in this review, comparators (C) will be Acute Covid (≤4 weeks from onset of symptoms) and Long Covid (>4 weeks from onset of symptoms), and outcomes (O) will be HRQoL of Covid-19 on health-related quality of life of patients (measured in physical, psychological, emotional and social dimensions), which were measured by different generic and specific tools, such as EQ-5D, SF-36, SF-6D (derived from SF-36), HUI (Health Utility Index), SGRQ. In this review, Covid-19 ‘confirmed patients’ are defined as those patients who are diagnosed with Covid-19 infection and confirmed by laboratory test (antigen or antibody). ‘Suspected patients’ are those patients with symptoms of Covid-19 who could not get confirmation because of a variety of reasons (e.g. unavailability of testing facilities, or unable to carry out the test), and who were self-isolated.

**Table 1:** Eligibility criteria.

Inclusion criteria	Exclusion criteria
1. Studies conducted on impact of Covid-19 on health-related quality of life of lab confirmed or suspected Covid-19 patients, with all levels of severity of illness, symptomatic patients	1. Studies not related to impact of Covid-19 on health-related quality of life of confirmed or suspected Covid-19 patients
	2. Studies which reported about the quality of life of general people, or certain patients group
2. Any types of original studies (case control, cohort, observational, cross-sectional, longitudinal, randomized control trials)	3. Studies not related to human
	4. Studies related to epidemiology only or molecular biology only
3. Studies on human, all ages, sexes and infected by Covid-19	5. Reviews, letters, communications, notes, editorials and conference reports
4. Studies published in English language	6. Study related to animals
5. Studies published in 2019 and Jan 2021	7. Not published in English language
	8. Studies published before December 2019

### Study selection, data extraction, analysis and reporting

**Fig 1:**
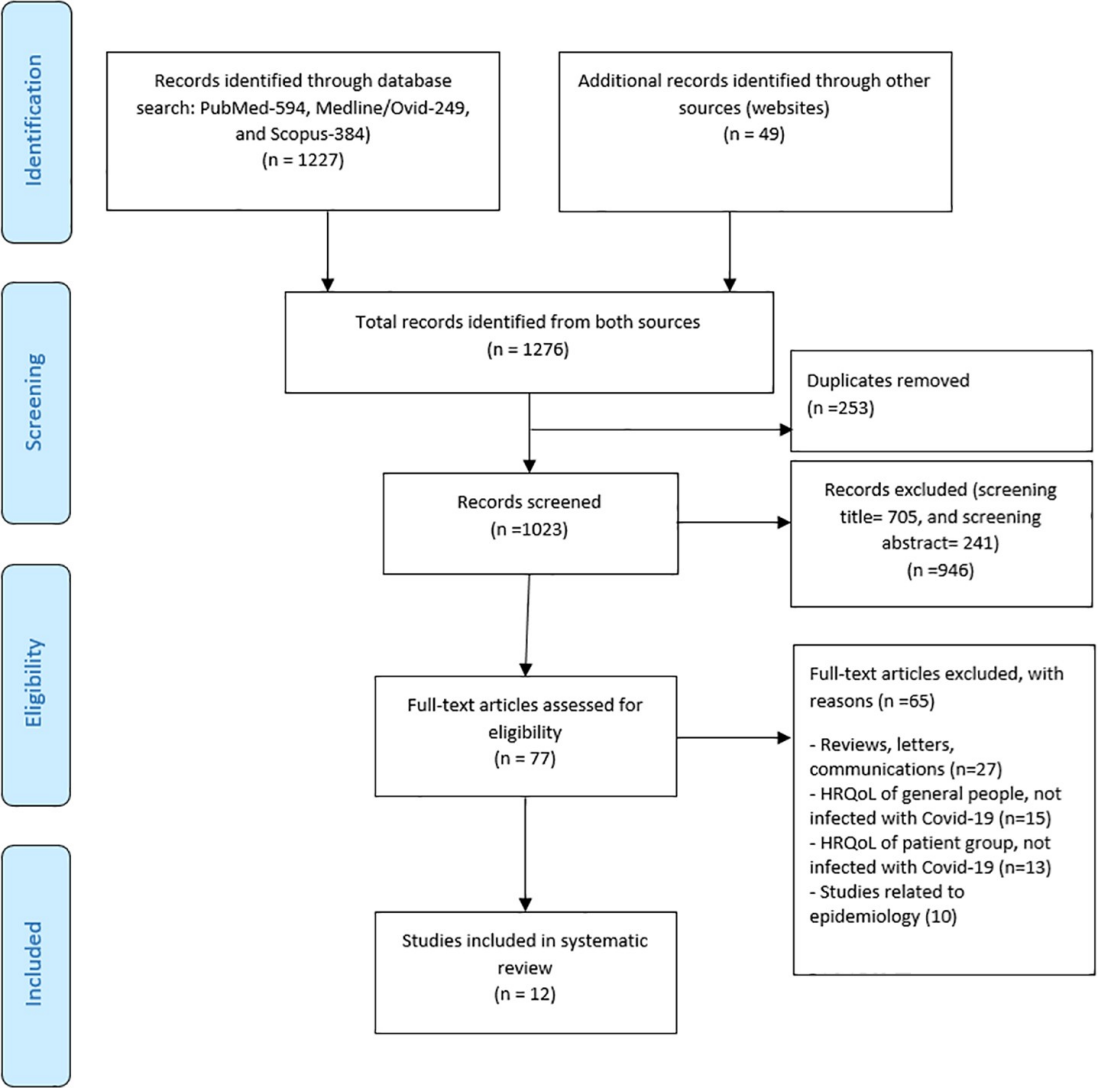
PRISMA 2009 flow diagram.

Studies in this review was selected by two reviewers (ANP and SZ) independently using eligibility criteria. Disagreement were discussed by third author (GLY) and resolved. Detailed study selection processes are presented in Fig 1. Data extraction was also done by two reviewers (ANP and SZ) using an Excel spreadsheet. The third author (GLY) checked the completeness of the data extraction and suggested additions where necessary. The form was piloted using three eligible papers and revised before use. The following information were extracted from the selected studies: first author and publication year, study title, study type (e.g. survey, observational or experimental studies), study country, sample size (male/female), age of patients (e.g. mean, median, range), Covid-19 confirmed or suspected cases, how data were collected, severity of the patients, hospitalised or non-hospitalised patients, tools used to assess the quality of life (e.g. SF-36, SF-6D, EQ-5D-5L, HUI, SGRQ), and the time point at which HRQoL data were collected (assessment time from the onset of symptoms). In addition, we extracted data on health-related HRQoL (mean, median, IQR, SD, percentage, frequencies, p values, etc.), and also statistically significant factors identified in the study as affecting HRQoL of patients due to Covid-19.

Based on literature, we categorised Covid-19 into ‘Acute Covid’ (AC) and ‘Long Covid’ (LC). Covid-19 is defined as ‘Acute’ when the symptoms last ‘up to 4 weeks’ from its onset [28], and it is defined as ‘Long’ if the symptoms last ‘more than 4 weeks’ [29, 30]. For those studies which did not clearly report their assessment time from the onset of symptoms (e.g. one month after discharge), we estimated the assessment time considering average length of stay reported in that study. Factors affecting Covid-19 on HRQoL on both groups of patients (i.e. Acute and Long Covid) are also assessed.

We conducted descriptive synthesis and analysis of the data in this review but did not perform meta-analysis because of the limited number of studies and heterogeneous nature of the data. The study outcomes of interest were the impact of Covid-19 on HRQoL of Acute Covid patients and Long Covid patients and factors affecting these outcomes. Data were extracted on mean or median values of all HRQoL variables (such as ‘pain/discomfort’, ‘self-care’) including respective standard deviation or 95% confidence interval, or interquartile range were reported, as it is important to know spectrum of HRQoL impacts. We estimated confidence intervals for studies in cases where this information was missing (e.g. Halpin et al. 2020), based on the included studies [31]. Where combined HRQoL scores of all patients were not given, separate index values of each group (e.g. male and female patients, ward and ICU patients) were calculated as the weighted mean (e.g. for study by Halpin et al. 2020) [32]. References were managed in EndNote and the report was prepared in line with PRISMA guidelines.

## Results

Fig 1 shows a flow diagram showing a total of 1227 studies were identified from database searches (PubMed, Medline and Scopus) and 49 studies were obtained from the website search. After removing duplicates, we retained 1023 studies. After screening by title and abstract, we retrieved 77 studies for full-text reading. After assessing the full-text, only 12 studies met our inclusion criteria and were therefore included in this review (no eligible studies were obtained from reference lists). Summary of these studies are provided in Table 2. Out of these 12 studies, the majority (n = 11) were observational (e.g. cross-sectional surveys) and one was an experimental study. The majority of the studies (n = 10) used generic HRQoL assessment tool (five used SF-36, five EQ-5D-5L), and the rest used a pulmonary disease-specific HRQoL tool, i.e. SGRQ (St George’s Respiratory Questionnaire) tool (2/12),Clinical COPD Questionnaire (CCQ) (1/12), and PROMIS tool (1/12) (2 out of the 12 studies used two HRQoL assessment tools i.e. SGRQ and EQ-5D-5L, and CCQ and EQ-5D-5L). Among 12 studies, nine studies included all confirmed Covid-19 cases, two studies included a mix of both confirmed and suspected cases and one study included all suspected cases. Likewise, nine out of 12 studies included hospitalised or previously hospitalised patients, two studies included non-hospitalised patients and one study included a mix of hospitalised and non-hospitalised patients. Three studies were conducted on Acute Covid patients (≤4 weeks from onset of symptoms) and 11 studies covered Long Covid patients (10 studies 4–12 weeks; 1 study >12 weeks) (HRQoL was assessed in both Acute and Long Covid patients in two studies, therefore total is >12).

**Table 2:** Summary of the studies included in the review.

First author, publication year	Country, study type, tools used to assess HRQoL	Sample size (male, female), % of confirmed cases	Age, illness severity, hospitalised or not	Assessment time from onset of symptoms, how patients were recruited	Major outcomes (HRQoL and Factors affecting HRQoL)
Nguyen et al. 2020 [23]	*Vietnam*	*3947 (with suspected Covid-19 symptoms: 1387; without suspected Covid-19 symptoms: 2560,	*Age 18 yrs and over (range 18 yrs to 85 yrs)	* HRQoL was assessed around 2 weeks from onset of symptoms	**HRQoL (SF-36) score:**
	*Cross-sectional study	*Male 1747, female 2197)	* Patients were not in emergency conditions	* Patients recruitment was done during outpatient visit	Without suspected Covid-19 symptom (mean, SD): 73.6 (15.2); With suspected Covid-19 symptoms 62.1 (18.8), Significant test: p<0.001
	*36-Item Short Form Survey (SF-36)	*With suspected cases vs without suspected cases	*All not hospitalised cases		**Multivariate analysis:** HRQoL score was significantly lower in the people aged 60 years or older (regression coefficient (B), -3.60; 95%CI, -5.13, -2.08, p < 0.001), with comorbidity (B, -2.81; 95%CI, -4.18, -1.45, p < 0.001); HRQoL score was significantly higher in men (B, 1.89; 95%CI, 0.82, 2.95, p = 0.001), in people with higher education attainment (B, 6.82; 95%CI, 4.85, 8.78, p < 0.001, in people with their own business (B, 2.25; 95% CI, 0.73, 3.77, p = 0.004), in people with middle or high social status (B, 4.62; 95%CI, 3.09, 6.15, p < 0.001), in people who did not drink (B, 1.74; 95%CI, 0.61, 2.87, p = 0.003), and in those who did more physical activity (B, 2.72; 95%CI, 1.52, 3.92, p < 0.001)
Chen et al. 2020 [33]	*China	*361 (male 186, female 175)	* Age 10 to 89 (mean 47.22, SD 13.03)	*Assessed around 6 weeks from onset of symptoms	**HRQoL (SF-36) score after one month of discharge:**
					**Physical functioning (PF) (mean, SD):** male patients: 95.13 (9.11), female (n = 175): 93.17 (10.26)
					**Role limitation due to physical problem (RP):** male 71.37 (34.73), female 72.29 (36.40)
	*Cross-sectional study	*All confirmed cases	*90.6% were mild cases	* Patients recruitment was done during first outpatient visit (at first month follow up)	**Bodily pain (BP):** male 95.59 (10.36), female 91.95 (16.49) **General health (GH):** male 78.31 (17.37) female 77.80 (19.01)
					**Vitality (VT):** male 83.25 (16.13), female 81.80 (16.32) **Social functioning (SF):** male 70.44 (27.68), female 64.66 (27.16)
					**Role limitation due to emotional problem (RE):** male 74.53 (40.54), female 66.64 (45.62) **Mental health (MH):** male 81.27 (27.46), female81.24 (17.37)
	*SF-36		*All previously hospitalised		Age, female sex, severity of illness, Length of stay (LOS), lung function were negatively associated (P<0.05) with most of the HRQoL dimensions; Overweight or obese were significant factors associated with a poor PCS score; female sex was a significant determinant associated with an MCS < 50 in Covid-19 patients.
Liu et al. 2020 [34]	*China, *prospective, quasi-experimental study	*72 (male 49, female 23); 36 with respiratory rehabilitation program and 36 without it)	*Age 65 yrs and above	* HRQoL assessed around 2 weeks and 8 weeks from onset of symptoms	**HRQoL (SF-36) score:**
					**Physical health (mean, SD):** 6 weeks ago 53.2 (7.7) vs after 6 weeks 54.1 (7.5)
					**Body role function:** 6 weeks ago: 61.3 (7.2) vs after 6 weeks 62.0 (7.3)
	*SF-36	*All confirmed cases	*Mean (SD) age 68.9 (7.6)	*Study conducted during hospitalisation	**Physical pain:** 6 weeks ago: 63.5 (8.1) vs after 6 weeks 62.9 (7.9)
					**General health**: 6 weeks ago: 61.8 (8.4) vs after 6 weeks 61.4 (6.9)
			*Severity not reported		**Energy or Vitality:** 6 weeks ago: 60.5 (7.1) vs after 6 weeks 61.2 (6.3)
					**Social function:** 6 weeks ago: 59.5 (7.0) vs after 6 weeks 58.9 (6.6)
			*All hospitalised patients		**Emotional role function:** 6 weeks ago: 61.4 (7.3) vs after 6 weeks 60.8 (7.3)
					**Mental health**: 6 weeks ago: 61.6 (7.2) vs after 6 weeks 62.1 (7.6)
Guo et al. 2020 [13]	*China, Questionnaire survey, Short Form 36 (SF-36)	*254 (male 119, female 135)	* Age 18 yrs or over * Mean, SD of age not reported	*HRQoL assessed around 6 weeks from onset of symptoms	**HRQoL (SF-36) score (after one month of discharge):**
					**Vitality (Median, IQR):** female 85 (77.5–92.5) vs male 90 (75–92.5), p = 1
			*Age group: 18–46 yrs: 130; 46–69 yrs: 106; >69 yrs: 5		**Social functioning (Median, IQR):** female 66.6 (44.44–88.8) vs male 77.7 (44.44–100), p = 0.38
		* All confirmed cases	*Patients without comorbidities, but all previously hospitalised	*Questionnaire survey conducted	**Role-emotional (Median, IQR):** female 100 (0.0–100) vs male 100 (66.6–100), p = 0.4
					**Mental health (Median, IQR):** female 84 (74–96) vs 84 (74–92), p = 0.12
					[They did not report other items]
					**Factors affecting HRQoL:** aged 46 to 69 yrs (p = 0.0472), positive nucleic acid duration longer than 14 days (p = 0.0311)
van den Borst et al. 2020 [35]	*Netherlands *Prospective observational study	*124 (male 74, female 50)	* Mean age 59, SD 14 yrs	*HRQoL assessed around 13 weeks from onset of symptoms	**HRQoL (SF-36) score (3 months after recovery):** Scores on all domains of SF-36 were low mainly- in role functioning, energy/fatigue and general health, most reported domains: fatigue (69%), functional impairments in daily life (64%), general quality of life (72%)
	*SF-36	* Confirmed and clinically suspected cases with > 6 weeks of symptoms	* 37% severe or critical patients rest mild or moderate	* All patients were invited at outpatients after 3 months of acute Covid-19 recovery	
			*Both hospitalised and non-hospitalised		
Daher et al. 2020 [18]	*Germany *Prospective study	*33 (male 22, female 11)	*Age mean (SD): 64 yrs (3)	*HRQoL assessed around 8 weeks from onset of symptoms	**HRQoL (EQ-5D-5L) score (after 6 weeks of discharge):**
					**EQ-5D-5L score (median, IQR):**
	*EQ-5D-5L (Euro Quality of life—five Dimensions—five Levels)	* All confirmed cases	* All patients had severe disease	* Those who came for follow up at 6 weeks after discharge were recreuited in the study	Mobility (walking): 2 (1–3)
			*All hospitalised before but discharged 6 weeks ago		self-care: 1(1–1)
					usual activities: 2(1–3)
					pain/discomfort: 2(1–3)
					anxiety/depression: 2(1–2)
					EQ VAS: 63(53–80)
					**SGRQ scores (median, IQR):**
	*St. George’s Respiratory Questionnaire (SGRQ)				Symptoms score: 34 (9–57)
					Activity score: 54(19–78)
					Impacts score: 12(2–33)
					Total score: 26 (7–42)
Arab-Zozani et al. 2020 [36]	*Iran	*409 (male 247, female 162)	*Mean age: 58.4 ± 18.21 years	*HRQoL assessed around 4–6 weeks from onset of symptoms	**HRQoL (EQ-5D-5L) score:**
	*Cross-sectional				
	study	*All confirmed cases	*Age groups: ≤40: 6.6%, 41–50 yrs: 26.4%, 51–60 yrs: 40.84%, >60: 26.16%	* Telephone interview was conducted	**% of patients reporting no problems (healthy state):** mobility 53.34%, self-care 87.75%, usual activities 58.97%, pain/discomfort 57.97% and anxiety/depression 41.26%. (Patients only reported unable to/extreme problems for the anxiety dimension). **The mean EQ-5D-5L index values (mean, SD):** 0.6125 ± 0.006. **Factors affecting HRQoL (univariate analysis):** gender (male 0.628 (0.201) vs female 0.585 (0.198), p = 0.002), age (<40 yrs 0.585 (0.198) vs >40 yrs (0.554 (0.145), p = 0.005), ICU 0.581 (0.201) vs Non-ICU 0.613 (0.167) (p<0.001), education status (p < 0.001), employment status (p < 0.001), and workplace status (p = 0.002), diabetes (p < 0.001), heart failure (p = 0.002), admitted to hospital (p < 0.001)
	*EQ-5D-5L		*All patients previously hospitalised		
*18% admitted in ICU					
Halpin et al. 2020 [32]	*UK	*100 (male 54, female 46)	* Age 18 yrs or over * Age (median, range) in years: Ward patients 70.5 yrs (20‐93); ICU patients 58.5 yrs (34‐84)	*HRQoL assessed around 6–10 weeks from onset of symptoms	**HRQoL (EQ-5D-5L) score (follow up interview at 4 weeks to 8 weeks):**
					**Ward patients (n = 68) vs ICU Patients (n = 32)** **Mean EQ-5D-5L (mean):** ward patients 0.724, ICU patients 0.693
	*Telephone interviews	*All confirmed cases		* Telephone interview was conducted	Worsened mobility: ward patients 30.9%, ICU 50%
					Worsened self-care: ward patients 17.6%, 12.5%
					Worsened usual activities: ward patients 36.8%, ICU patients 29.4%
	*EQ‐5D‐5L		*All patients previously hospitalised		Worsened pain/discomfort: ward patients 14.7%, ICU patients 28.1%
					Worsened anxiety/depression: ward patients 16.2%, ICU patients 37.5%
			*32% participants admitted in ICU			Post discharge symptoms prevalence (%):
					Any new fatigue: ward patients 60.3%, ICU patients 72%
					Any new or worsened breathlessness: ward patients 42.6%, ICU patients 65.6%
					Any PTSD symptoms related illness: ward patients 23.5%, ICU patients 46.9%
Lerum et la. 2020 [37]	*Norway *Prospective cohort study	*103 (male 54, female 49)	*Age 18 years or above	*HRQoL assessed around 8 weeks from onset of symptoms	**HRQoL (EQ-5D-5L) score (at 3 months follow up visit (:**
	*EQ-5D-5L	* All confirmed cases	*Median age (IQR) yrs: 59 (49, 72)	* Eligible patients were invited by mail about six weeks after hospital discharge and recruited	Participants admitted to ICU had a higher median score on usual activities than participants admitted to regular wards only, 4 (25-75th percentile 2–4) vs 2 (1–2), respectively (p = 0.014)); median EQ-5D index scores (SD) were 0.61 (0.23) and 0.72 (0.19) for ICU and non-ICU patients,(p = 0.087)
			*All hospitalised before but discharged at the assessment time		
Meys et al. 2020 [17]	*Belgium	*210 (male 26, female 184)	* Aged 18 years and over	*HRQoL assessed around 10–12 weeks from onset of symptoms	**HRQoL (EQ-5D-5L) score (after 79+ - 17 days from onset of symptoms):**
	*Cross-sectional study	*Covid-19 confirmed or suspected cases	* Data of 210 non-hospitalized patients (88% women, age 45 (11) years)	* Completed an online survey	EQ-5D index score (mean, SD): 0.62 (0.19),
	*EQ-5D-5L				* All non-hospitalised patients	**EQ-5D-5L dimension scores (mean, SD)**
	*Clinical COPD Questionnaire (CCQ)					Mobility: 2.41±1.04
						Self-care: 1.19±0.54
						Usual activities: 2.95±0.98
						Pain/Discomfort: 2.87±0.77
						Anxiety/Depression: 2.10±0.96
						40% of the patients had EQ-5D index below the fifth
						percentile; mean EQ-VAS was 50.7 (18.9) (range: 0–99); usual activities &
						pain/ discomfort dimensions showed the greatest self-reported impairment: 67% & 70% reported at least moderate problems, respectively; self-care dimension—86% reported no problems;
						**HRQoL (CCQ) Scores:**
						Mean (SD) CCQ score: 2.01 (0.98) points; symptoms & functional state domains were equally affected (mean scores 2.13 (1.12), 2.12 (1.22), respectively); CCQ items 2 (64% patients having shortness of breath during physical activities) & 7 (70% patients least moderately limited due to their respiratory symptoms during strenuous physical activities) had the greatest impact on total scores
Santus et al. 2020 [10]	*Italy	*20 (17 male, 3 female)	* Mean age of 55 years (SD 15)	*HRQoL assessed around 2 weeks and after 4 weeks (32–33 days) from onset of symptoms	**HRQoL (SGRQ) score:**
					(T0: at hospital discharge, T1: at 15 days after hospital discharge)
	*Observational study	*All confirmed cases	* All hospitalised patients		**Mean (SD) SGRQ total score:** At T0 25.5 (15.5) vs at T1 16.9 (13.2) (p<0.01)
					**Mean SGRQ symptoms score:** At T0 33.7 (18.0) vs at T1 16.7 (12.9) (p<0.01)
					**Mean SGRQ activity score:** At T0 35.7 (24.2) vs at T1 28.3 (23.3) (p<0.01)
	*St George’s Respiratory Questionnaire (SGRQ)		* All discharged from high dependency unit	*Questionnaires were completed by participants at the time of hospital discharge and on Day 15 post-discharge	**Mean SGRQ impact score:** At T0 17.3 (15.9) vs at T1 10.6 (10.7) (p<0.01)
					(65% of patients achieved a clinically significant improvement in the SGRQ total score between hospital discharge and Day 15, and 30% of patients achieved a clinically significant improvement in the mMRC dyspnoea scale)
Jacobs et al. 2020 [38]	*USA	*183 (male 112, female 71)	*Aged 18 or older; *Median age was 57 years (interquartile range [IQR] 48–68; range 25–85)	*HRQoL assessed around 6 weeks from onset of symptoms	**HRQoL Score (PROMIS) (at 35 days after (±5) discharge):**
	*Prospective cohort study,	*All confirmed cases	*All previously hospitalised,	* Questionnaire was administered by email (23%) or telephone call (48%) to participants	**At 35 days after discharge (+ - 5):** General health: poor, fair- 20.2%, good- 38.2%, very good/excellent- 41.5%; Quality of life: poor, fair- 23.2%, good- 37%, very good/excellent- 39.8%; Physical health: poor, fair-27.1%, good- 34.2%, very good/excellent- 38.7%; Mental health: poor, fair- 16.9%, good- 39.3%, very good/excellent- 43.7%; Fatigue: severe, very severe: 8.2%, moderately: 32.8%, none, mild- 59% *Older participants aged 65 to 75 years (OR 8.666, 95% CI: 2.216, 33.884, p = 0.0019); women (male versus female gender OR 0.462, 0.225, 0.949 p = 0.0356]), had statistically significant higher odds of experiencing persistent symptoms; persistent symptoms were reported by 72.7% participants at day 35;
*PROMIS tool (v1.0 and v1.2-Global Health)	* 95.4% of all participants were classified as mild severity of illness				

### Health-related quality of life measured by SF-36

Five out of twelve studies used SF-36 tool to assess the HRQoL of patients affected by Covid-19 [13, 23, 33, 34]. A 36-item Short-Form (SF-36) health survey is a generic instrument (which does not use a preference based approach) to assess the HRQoL, which is used in clinical practice and research, health policy and evaluations and general population surveys [39]. It assesses eight health concepts (score range from zero to 100, a score of zero is equivalent to maximum disability and a score of 100 is equivalent to no disability): physical functioning (PF), role physical (RP), bodily pain (BP), general health (GH), vitality (VT), social functioning (SF), role emotional (RE) and mental health (MH) [39, 40]. The majority of the included studies were conducted in China (3/5) and studies were mainly observational (4/5). Only three studies provided HRQoL scores of Covid-19 patients (using SF-36) [13, 33, 34]; one study provided combined (i.e. average total of all dimensions) HRQoL score (62.1±18.8) [23] and another study provided proportion of patients with functional and emotional impairment [35]. Amongst the three studies that reported HRQoL scores, only one study assessed HRQoL during Acute stage (<4 weeks) and after Acute stage, i.e. Long Covid (>4 weeks) [34]. The overall mean HRQoL scores of Acute Covid patients was 60.3 [34] and Long Covid patients ranged from 60.4 [34] to 86.4 [13], with higher SF-36 score representing better health (Table 3). The lowest HRQoL score (60.4) was among the elderly patients (aged over 65 years) and highest HRQoL score (86.4) was with the majority of younger patients (54%, 18–46 years) and all patients were without comorbidities.

**Table 3:** SF-36 components’ score reported in different studies (Acute Covid and Long Covid).

Author and publication year (Ref no.)	PF mean (SD) score	RP mean (SD) score	BP mean (SD) score	GH mean (SD) score	VT mean (SD) score	SF mean (SD) score	RE mean (SD)score	MH mean (SD) score	Overall mean (range) score**
**Acute Covid (≤4 weeks from onset of symptoms)**									
Liu et al. 2020 [34]	53.2 (7.7)	61.3 (7.3)	63.5 (8.1)	61.8 (8.4)	60.5 (7.10	59.5 (7.0)	61.4 (8.4)	61.6 (7.2)	**60.3 (53.2–63.5)**
**Long Covid (>4 weeks from onset of symptoms)**									
Chen et al. 2020 (¥) [33]	95.1 (9.1)	71.4 (34.7)	95.6 (10.4)	78.3 (17.4)	83.2 (16.1)	70.4 (27.7)	74.5 (40.5)	81.3 (27.5)	**81.2 (70.4–95.6)**
Chen et al. 2020 (¥) [33]	91.2 (10.3)	72.3 (36.4)	91. 9 (16.5)	77.8 (19.0)	81.8 (16.3)	64.7 (27.2)	66.6 (45.6)	81.2 (17.4)	**76.5 (64.7–91.9)**
Chen et al. 2020 [33]#	94.2	71.8	93.8	78.1	82.5	67.6	70.7	81.3	**79.8 (67.6–94.2)**
Liu et al. 2020 [34]	54.1 (7.5)	62 (7.3)	62.9 (7.9)	61.4 (6.9)	61.2 (6.3)	58.9 (6.6)	60.8 (7.3)	62.1 (7.6)	**60.4 (54.1–62.9)**
Guo et al. 2020 [13] (¥)	-	-	-	-	90 (75–92.5)*	77.7 (44.4–100)*	100 (66.6–100)*	84 (74–96)*	**87.9 (77.7–100)**
Guo et al. 2020 [13] (¥)	--	-	--		85 (77.5–92.5)*	66.6 (44.4–88.8)*	100 (0.0–100)*	84 (74–92)*	**83.9 (66.6–100)**
Guo et al. 2020 [13]#	-	-	-	-	87.3	71.8	100	84	**86.4 (71.8–100)**

[Note: Two studies [23, 35] presented results in different style, therefore not suitable to present in the above table].

PF- physical functioning, RP- role physical, BP- bodily pain, GH- general health, VT- vitality, SF- social functioning, RE- role emotional and MH- mental health.

*Median and interquartile range.

** Calculated average scores for the review (i.e. not reported in the papers).

# Calculated weighted average from male and female patient’s groups (only average of male and female patients reported in the study).

¥HRQoL score of male patients only.

ⴕ HRQoL score of female patients only.

**Fig 2:**
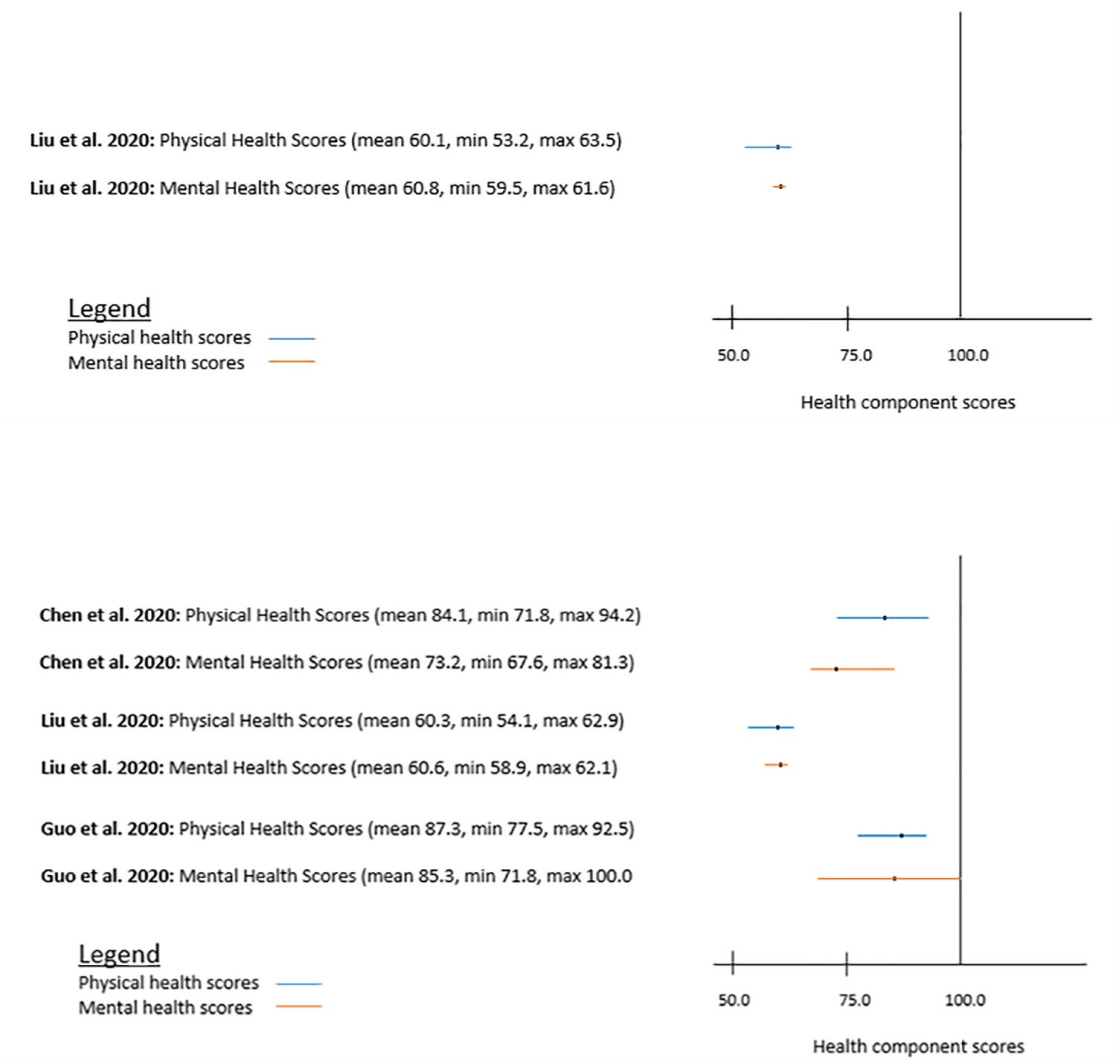
a. SF-36 physical and mental health components scores of Acute Covid. b. SF-36 physical and mental health components scores of Long Covid.

In the Acute Covid, highest score was reported in bodily pain (63.5) and lowest score was reported in physical functioning (53.2). However, in Long Covid highest score was reported in different dimensions in different studies. For example, Chen et al. (2020) reported highest score on physical functioning (94.2) [33]and Guo et al. (2020) reported the highest score on role emotional (100%) [13]. Likewise, similar variations were found in the lowest score on different dimensions, meaning there were no pattern on the highest or lowest scores based on HRQoL dimensions (Table 3). In Long Covid, mean physical components scores (PCS) were slightly higher than mental components scores in general (Fig 2). It is not possible to compare SF-36 HRQoL scores by country because of heterogeneity in presenting results and all three studies were from one country (i.e. China) (other two studies reported outcomes differently, not suitable for comparison). Likewise, impact of Covid-19 on HRQoL using SF-36 was not reported by illness severity to compare with. Only two study provided HRQoL scores by gender [13, 33] and overall mean scores were higher in male patients (81.2 to 87.9) than female patients (78.7 to 83.9) in both studies [13, 33] (Table 3).

According to a study on Acute Covid (≤4 weeks) [23], HRQoL score was significantly lower in the people aged 60 years or older (regression coefficient (B), -3.60; 95%CI, -5.13, -2.08, p < 0.001), and with comorbidity (B, -2.81; 95%CI, -4.18, -1.45, p < 0.001). HRQoL score was significantly higher in men (B, 1.89; 95% CI, 0.82, 2.95, p = 0.001), in people with higher education attainment (B, 6.82; 95% CI, 4.85, 8.78, p < 0.001, in people with their own business (B, 2.25; 95% CI, 0.73, 3.77, p = 0.004), in people with middle or high social status (B, 4.62; 95% CI, 3.09, 6.15, p < 0.001), in people who did not drink (B, 1.74; 95% CI, 0.61, 2.87, p = 0.003), and in those who did more physical activity (B, 2.72; 95% CI, 1.52, 3.92, p < 0.001) [23].

A study about Long Covid (> 4 weeks) reported that factor affecting HRQoL score was positive nucleic acid duration (longer duration had lower RE) was a factor affecting RE negatively (p = 0.01) [13]. Likewise, VT and MH scores were significantly affected by positive nucleic acid duration (longer than 14 days, p = 0.0311) and age group (age 46–69 years, p = 0.0472) [13]. Another study [33] showed that age was negatively associated with PF, RP (p<0.05), as reported above. PF, BP, and RE were negatively associated with the female sex (p< 0.05). Length of stay (LOS) was negatively associated with RE and RP. Likewise, there were significant negative relation between lung function (Forced vital capacity, FVC) and mental health dimension (MH) (P<0.05). Logistic regression analysis demonstrated that being overweight (OR 3.71, 95% CI 1.42–9.70) or obese (OR 3.94, 95% CI 1.47–10.52) were significant factors linked with a poor physical component summary (PCS) score. Female gender (OR 2.22, 95% CI 1.30–3.81) was a significant determinant associated with a mental component summary (MCS) (< 50) in COVID-19 patients.

### Health-related quality of life measured by EQ-5D-5L

Five out of twelve studies used EQ-5D-5L tool to assess the HRQoL of patients with Covid-19 in this review [17, 18, 32, 36, 37]. EQ-5D-5L is a generic and preference based HRQoL instrument for describing and valuing health and higher index value represents a better health. It is based on a descriptive system that defines health in terms of five dimensions: Mobility, Self-Care, Usual Activities, Pain/Discomfort, and Anxiety/Depression [41]. A single utility score can be generated from the five dimensions questionnaire based on published tariffs with value 0 for death and 1 for perfect health. Negative value indicates life worse than death [41, 42].

**Fig 3:**
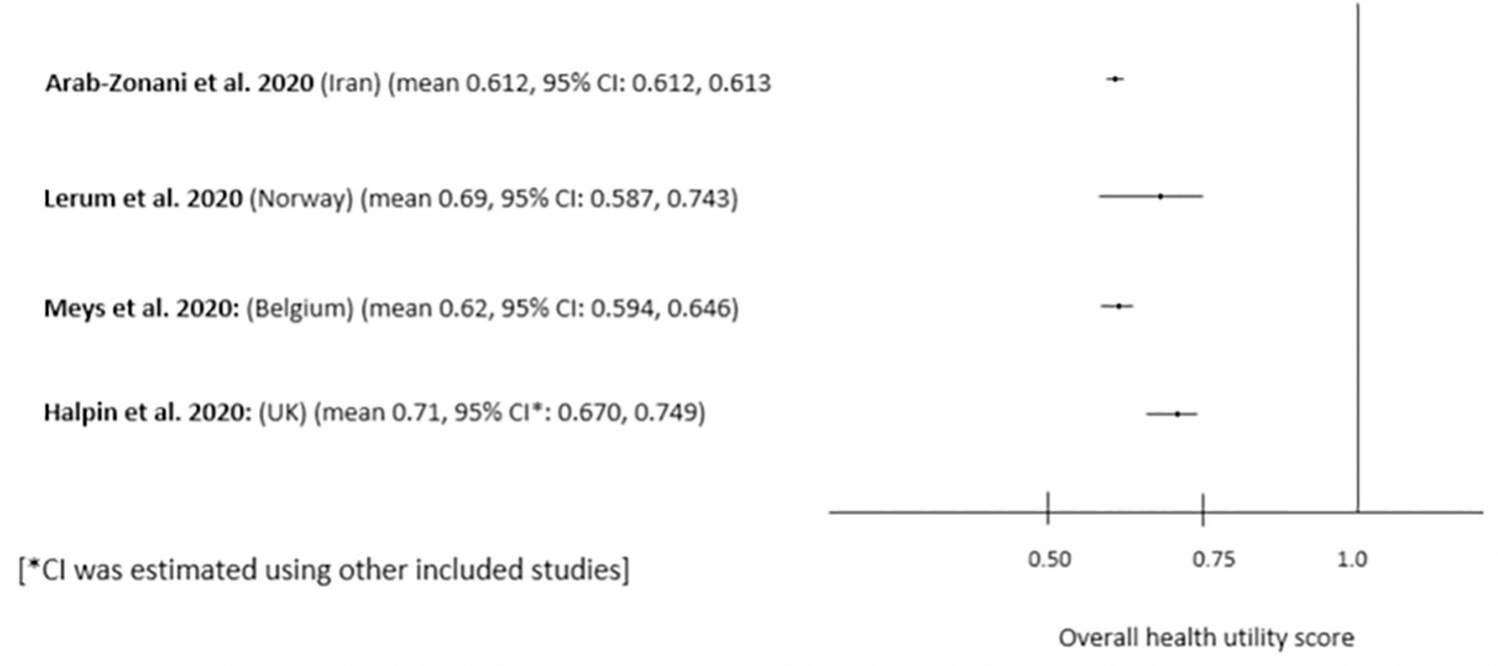
Mean EQ-5D-5L scores of Long Covid reported in different studies. [Note: Only those studies that reported mean EQ-5D-5L scores were presented in the Fig 3].

All five reviewed studies were observational, focused on Long Covid (>4 weeks) and conducted in Belgium, Germany, Iran, Norway and the United Kingdom. Mean HRQoL values in four studies (from Belgium, Iran, Norway, and the UK) were reported in a similar way, but the fifth study presented results in a different style [18]. The highest EQ-5D-5L index mean value was reported in the UK (0.714) [32], followed by Norway (0.690) [37], Belgium (0.620) [17] and lowest in Iran (0.612) [36] (Fig 3). The study in Iran covered all patients same as in the UK, but time of HRQoL assessment was roughly two to four weeks earlier in Iran (4-6weeks from onset of symptoms) than in the UK (6–10 weeks from onset of symptoms). It can be confirmed that time of HRQoL assessment is not the sole factor affecting HRQoL score because the study in Belgium [17] reported lower score than in the UK and Norway although it was assessed two to four weeks later (10–12 weeks after the onset of symptoms) than those studies covering similar patients in the UK and Norway (Fig 3).

**Fig 4:**
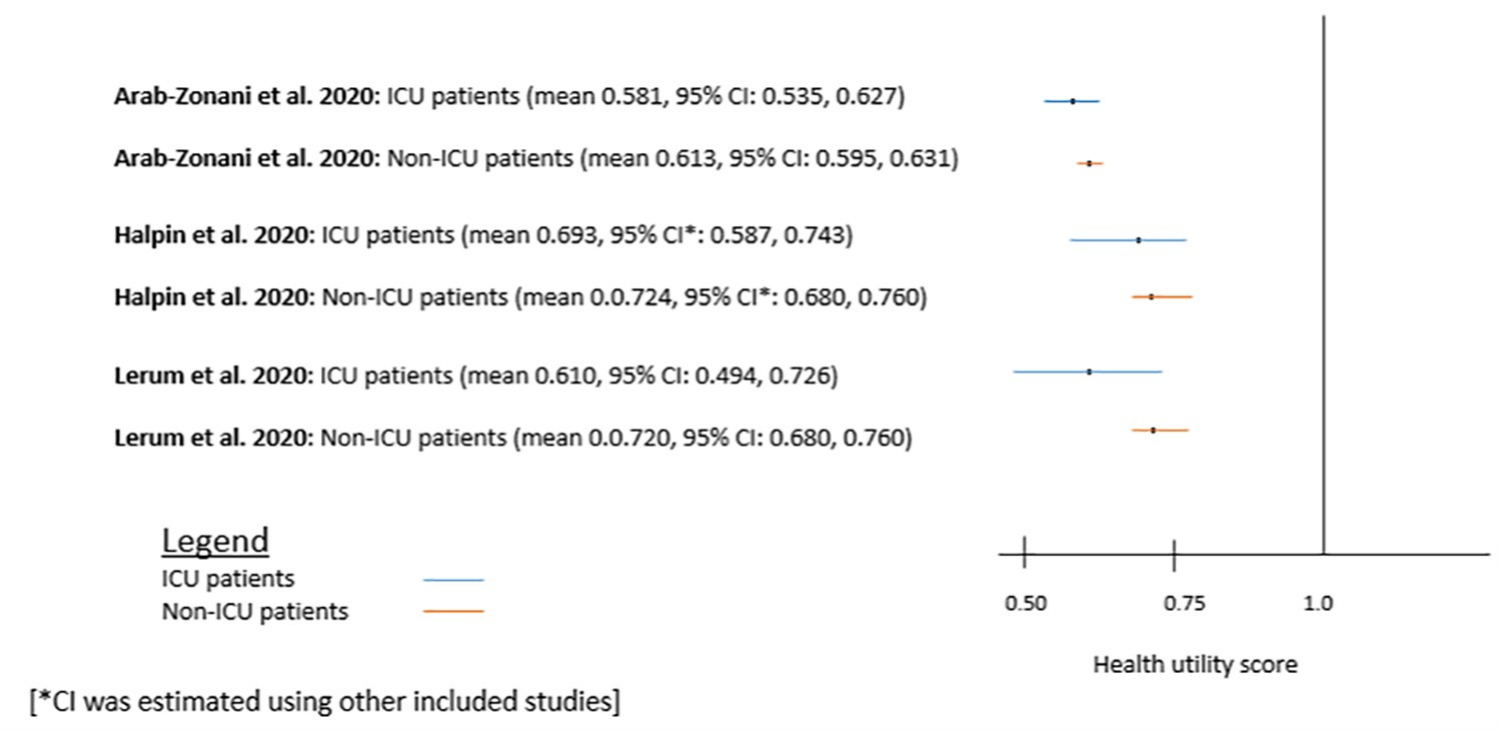
Mean EQ-5D-5L scores for ICU and non-ICU patients reported in different studies.

EQ-5D-5L scores for various dimensions were reported differently, making it difficult to present the pooled estimates for these dimensions. However, three out of five studies (Table 4) provided percentage of Covid-19 patients reporting difficulties for different dimensions of EQ-5D-5L [17, 32, 36]. Table 4 shows that overall difficulties (average %) the patients are facing, are comparable across three studies (25.9% to 45.2%). However, highest mobility problem was reported by the study in Iran (46.7%), self-care in the UK (16.0%), usual activities and pain/discomfort in Belgium (67.0% and 69.6% respectively), and anxiety/depression in Iran (58.7%) (See Table 4 for detail). Two studies [17, 18] reported EuroQoL Visual Analogue Scale (EQ VAS) scores 63 reported by Daher et al. (2020) in Germany [18] and 50.7 reported by Meys et al. (2020) in Belgium [17] (Table 2). The review found the higher HRQoL score for: non- ICU patients compared to ICU patients (Fig 4), male patients (0.63±20) compared to female patients (0.58±20), and younger patients (age ≤40 yrs) (0.62±0.32) compared to older patients (age ≥40) (0.55±0.15) (Table 2, study by Arab-Zozani et al. 2020) [36].

**Table 4:** Percentage of Long Covid patients reported problems in different dimensions of EQ-5D-5L.

Author and publication year	Mobility (walking) (%)	Self-care (%)	Usual activities (%)	Pain/ discomfort (%)	Anxiety/ depression (%)	Overall percentage* (%)
Arab-Zozani et al. 2020 [36]	46.7	12.3	41.0	42.0	58.7	40.1
Halpin et al. 2020 [32]	37.0	16.0	34.4	19.0	23.0	25.9
Meys et al. 2020 [17]	46.2	14.4	67.0	69.6	29.0	45.2

[Note: Two studies [18, 37] presented results in different style, therefore, not suitable to present in the above table. There was no study on Acute Covid patients using Eq-5D-5L tool].

*Calculated average % based on the report (not reported in the papers).

A study about Long Covid (> 4weeks) in Iran [36] reported that the EQ-5D-5L index score was significantly different (in univariate analysis) by gender (p = 0.002) (males had higher score), age (p = 0.005) (higher in age ≤ 40 years), educational status (p<0.001) (higher in patients with higher educational status), employment status (p < 0.001) (higher in patients with employment), and workplace status (p = 0.002) (higher in uncrowded workplace). Among the clinical factors (from univariate analysis), the mean EQ-5D-5L index score was significantly lower if the patient had diabetes (p < 0.001), or heart failure (p = 0.002) or was admitted to hospital (p < 0.001) [36].

### Health-related quality of life measured by SGRQ

There were two studies which assessed HRQoL of patients using St George’s Respiratory Questionnaire (SGRQ) [10, 18], which is pulmonary disease specific quality of life assessment tool. The score for each domain and the total score are ranged from 0 (no impairment/ no effect on quality of life) to 100 (maximum impairment/ maximum perceived distress). Thus, a higher score represents greater impairment or a poorer HRQoL. Both were observational studies and conducted in Italy and Germany. One of the studies compared HRQoL scores at two points of time: at the time of discharge from hospital and at 15 days of discharge [10].

Table 5 reports the SGRQ score on different dimensions among Acute Covid and Long Covid patients. The study in Italy by Santus et al. (2020) [10] reported that the SGRQ scores are significantly decreased (i.e. improved health) (p<0.01) in each dimension by time (comparing the assessment between around 2–3 weeks and 4–5 weeks, i.e. Acute Covid and Long Covid). The study also reported that 65% of patients achieved a clinically significant improvement in the SGRQ total score between hospital discharge and Day 15 [10]. However, while looking at the study conducted by Daher et al. (2020) in Germany [18] (Table 5), the patients with Long Covid (8 weeks from symptoms onset), SGRQ scores are comparable to the Acute Covid reported by Santus et al. (2020). As we found in the study, all respondents included in Daher et al. (2020) study had severe illness due to Covid-19, unlike reported in the study by Santus et al. (2020).

**Table 5:** SGRQ scores reported in included studies (Acute Covid and Long Covid).

Author and publication year	Symptoms (range 0–100)	Activity (range 0–100)	Impact (range 0–100)	Total core (range 0–100)	VAS (range 0–10)
**Acute Covid (≤4 weeks)**					
Santus et al. 2020 [10]	33.7 (18.0)*	35.7 (24.2)*	17.3 (15.9)*	25.5*	1.6 (1.7)*
**Long Covid (>4 weeks)**					
Santus et al. 2020 [10]	16.7 (12.9)*¥	28.3 (23.3)*¥	10.6 (10.7)*¥	16.9 (13.2)*¥	1.4 (2.5)*¥
Daher et al. 2020 [18]	34 (9–57)^#^	54 (19–78) ^#^	12 (2–33) ^#^	26 (7–42) ^#^	-

* Scores were reported in mean and standard deviation (SD).

# Scores were reported in median and interquartile range (IQR).

¥ SGQ scores are significantly decreased (p<0.01) in each dimension among patients with Long Covid compared to the patients with Acute Covid in the study in Italy [10].

### Health-related quality of life measured by CCQ

The Clinical COPD Questionnaire (CCQ) is a 10-item respiratory-specific quality of life assessment tool, which is divided into three domains: symptoms, mental state and functional state. The main outcomes are the CCQ total score (total scores of all domains divided by 10) and mean scores of the three separate domains. The scores are ranged from 0 to 6 points, with a higher value indicating lower quality of life [43]. The Clinical COPD Questionnaire (CCQ) tool was used by a study conducted in Belgium [17] for the Long Covid patients and reported the mean CCQ score as 2.01 points (± 0.98). According to the study, the symptoms and functional state domains were equally affected (2.13 ± 1.12 and 2.12 ± 1.22 points, respectively). The mental state domain was less affected compared to symptoms and functional state (1.56 ± 1.31) [17]. CCQ items 2 (Shortness of breath doing physical activities: 3.24±1.80) and 7 (Strenuous physical activities: 3.41±1.74) had the greatest impact on total scores, with 64% of the patients having shortness of breath during physical activities and 70% had problems during strenuous physical activities, respectively [17].

### Health-related quality of life measured by PROMIS scale

PROMIS tool is used to identify symptoms and assesses the quality of life parameters [38]. This tool particularly assesses the general health, quality of life, physical health, mental health and social active role including fatigue, dyspnoea and muscular pain. A study in the USA [38] reported that 72.7% Covid-19 patients had persistent symptoms at 35 days after discharge, 55.0% reported fatigue, 50.6% muscular pain, 45.3% shortness of breath and 41.82% cough. Older patients aged 65 to 75 years [OR 8.666 (2.216–33.884), p = 0.0019] and women (male vs female: OR 0.462 (0.225–0.949), p = 0.0356), had significantly higher odds of experiencing persistent Covid-19 symptoms [38]. Covid-19 patients’ self-rated quality of life and activities of daily living scores at 35 day after discharge were: a lower odds rating general health (poor/fair 20.2%, OR 0.093 [95% CI: 0.026, 0.329], p = 0.0002), quality of life (poor/fair 23.2%; OR 0.116 [95% CI: 0.038, 0.364], p = 0.0002), physical health (poor/fair 27.1%, OR 0.055 [95% CI: 0.016, 0.193], p <0.0001), mental health (poor/fair 16.9%, OR 0.093 [95% CI: 0.021, 0.418], p = 0.0019) and social relationship (poor/fair 60.4%, OR 0.095 [95% CI: 0.031, 0.291], p<0.0001) [38]. Thus, even at 35 days after discharge, a considerable proportion of Covid-19 patients experienced persistence symptoms and poor quality of life.

## Discussion

Main findings of the review are- the HRQoL score (i.e. SF-36 score) of patients with Acute Covid found to be lower compared to the patients with Long Covid. In Acute Covid, mental components score was slightly higher than physical components score (PCS) [34], but opposite was found in Long Covid [13, 33]. The HRQoL scores of elderly patients were not considerably improved even after six weeks of discharge from hospitals [34]. The long-term impacts of Covid-19 is still in its initial stage and it has not been fully developed yet. Most of the available studies on impact of Covid-19 on HRQoL were conducted between 4 and 12 weeks from the onset of symptoms. There were some symptoms which were reported by patients with Long Covid found in our review include fatigue, muscular pain, shortness of breath and cough [35, 38]. There were differences in the impact of Covid-19 on HRQoL of patients by study country, i.e. better HRQoL of the patients with Covid-19 from HICs compared to LMICs [32, 36, 37]. The common factors causing impact on both Acute and Long Covid were age, gender, severity of illness, comorbidity, income and educational level of the patients.

As reported in the main findings above, overall HRQoL score of Acute Covid patients was lower (mean SF-36 score 60.3) compared to patients with Long Covid. However, HRQoL scores (using SF-36 tool) of Long Covid patients are still low and vary from 60.4 (lowest) to 86.4 (highest) [13]. The lowest HRQoL score in Long Covid patients in this review was mainly due to recruitment of only elderly Covid-19 patients group (aged >65 years) [34] unlike in other studies [13, 33]. In addition, the HRQoL scores were not considerably improved among certain patient groups (e.g. elderly, ICU admitted patients) even after six weeks of discharge and lower in some domains even after three months of recovery (fatigue 69%, functional impairments in daily life 64%, and general quality of life 72%) [35]. In line with our findings, a study in Brazil among general population (age ≥7 years) reported that all dimensions of quality of life (using SF-36) significantly reduced during Covid-19 isolation than before isolation (p<0.05) [21].

All reviewed studies using EQ-5D-5L were on Long Covid (4 to 13 weeks from the onset of symptoms) and the mean EQ-5D-5L index values ranging from 0.61 to 0.71, which are considerably lower than outcomes of a similar study conducted with general population in China during the pandemic (0.949, SD 0.102) [44] and in Morocco (0.86) [45]. Lower magnitude of difficulties were reported among general population during Covid-19 pandemic in Vietnam [46] than found in our review [18]. Using SGRQ tool, studies found that there were considerable negative effects on patients’ HRQoL mean score, ranging from 17.3 (SD: 15.9) to 35.7 (SD: 24.2) in Acute Covid and 10.6 (SD: 10.7) to 54 (IQR 19–78) in Long Covid for different dimensions of SGRQ. Higher SGRQ score (means lower HRQoL) in Long Covid in this review was mainly found in the study in Germany [18] because they included only severe patients in their study. Unlike shown by SF-36 and EQ-5D-5L, a study using SGRQ by Santus et al. (2020) showed significant improvement in HRQoL of patients even after two weeks of discharge (i.e. Acute Covid vs Long Covid) [10]. This may be because they included only those patients who were clinically stable and able to fill the questionnaire [10].

A study on Long Covid included in our review (Jacobs et al., 2020, USA) reported that 72.7% patients (95% CI: 65.6, 78.9) had persistent symptoms at day 35 after discharge and majority experienced fatigue (55%), muscular pain (50.6%), shortness of breath (45.3%) and cough (41.8%) [38]. These are similar to the findings reported in other studies [17, 32]. Although there were studies conducted on the impact of Long Covid on HRQoL (conducted between 4 and 13 weeks from onset of symptoms), longer-term effects of Covid-19 (beyond 13 weeks) has not been fully developed yet [9]. A report published by WHO discussed about possible long-term impact of corona virus [8]. They reported that people recover from the illness after two to six weeks. However, some symptoms may linger or recur for weeks or months. Some patients may develop medical complications that may have lasting health effects. There may be prolonged illness due to the virus in young adults and children without underlying chronic medical conditions. More research needed to understand the long-term effects of coronavirus, why symptoms persist or recur, how these health problems affect patients and the clinical course and likelihood of full recovery.

While comparing the HRQoL by study countries, the highest EQ-5D-5L index value was reported in the high income countries (HICs), like UK (0.714) [32] and Norway (0.690) [37], and lowest in the low-and middle-income country (LMIC), Iran (0.612) [36]. The higher HRQoL of Covid-19 patients in high income countries like in the United Kingdom and Norway may be due to better health services of the countries rather than other factors [47, 48] compared to LMIC, such as Iran. We confirmed from the study level investigation that patients’ characteristics were not the causal factors for better health of the patients from the UK and Norway and worse HRQoL from the patients of Iran. For example, mean age of patients in the study of Iran was 58.4 (SD 18.2) and 18% of these patients were admitted in ICU. In the study in the UK, median age of the patients were 70.5 (range 18–93) and 32% of the patients were admitted in ICU. This means, the lower HRQoL of patients in Iran was not due to patients’ characteristics, such as elderly patients or severity of illness.

The review also explored the factors affecting Acute Covid (≤4 weeks from onset of symptoms) from different studies. HRQoL score (using SF-36) was significantly different by age of patients (lower in the patients aged 60 years or older, p< 0.001) and comorbidity (patients with comorbidity, p< 0.001) [23]. This is similar to the finding reported in a study from Canada [49]. In addition, HRQoL score was significantly different by gender (higher in male patients, p = 0.001), educational status (higher in patients with higher education attainment, p<0.001), business status (in people with their own business, p = 0.004), ability to pay for medication (higher score with better ability to pay, p <0.001), social class (in people with middle or high social status, p<0.001), alcohol use (in people who did not drink, p = 0.003), and physical exercise (in those who did more physical activity, p<0.001) [23].

This review found that HRQoL of Long Covid patients (>4 weeks of onset of symptoms) admitted in ICU (severely ill patients) were worse EQ-5D-5L scores ranging from 0.581 to 0.693) than patients admitted in normal ward (moderately ill patients) (scores ranging from 0.613 to 0.724) [32, 36, 37]. This finding is intuitive as severity of illness impact on physical health, mental health and well-being of people and thus reduces quality of life [50]. Moreover, this review found HRQoL scores in male patients (SF-36 scores from 81.2 to 87.9) were higher than female patients (scores from 78.7 to 83.9) [13, 33] (while keeping other factors constant). These findings are also supported by other similar study [36]. The impact of Covid-19 was found worse in older patients (≥60 yrs: 0.554) than younger patients (≤40 yrs: 0.618). A study in the USA also supported above findings [38]. A study among general population during Covid-19 pandemic also reported that people with aging had lower HRQoL scores than younger population [44]. In addition, a study about Long Covid (> 4 weeks) (using SF-36) reported that factor affecting HRQoL score was positive nucleic acid duration (longer duration had lower RE) (p = 0.01) [13]. Length of stay (LOS) was negatively associated with RE and RP. Logistic regression analysis showed that being overweight (p<0.05) or obese (p<0.05) were significant factors linked with a poor physical component summary (PCS) [33]. These findings were also supported by a study in Morocco and Vietnam [45, 46].

Majority of the included studies covered impact of Covid-19 on hospitalised or previously hospitalised patients. There were lack of studies covering non-hospitalised patients with Covid-19. Likewise, most of the studies (10 out of 12, 2 studies covered both- both Acute and Long Covid) assessed the impact of Covid-19 on HRQoL between four weeks and 12 weeks from the onset of symptoms. There were lack of HRQoL studies conducted on Acute Covid patients within 4 weeks from onset of symptoms and on Long Covid patients after 12 weeks from onset of symptoms. Similarly, there were no studies, which assessed impact of Covid-19 on patients under 18 years of age. As we discussed in the results section, impact of Covid-19 on HRQoL of patients were mainly measured using generic instruments, such as SF-36 and EQ-5D, and none of the studies have reported its preference-based counterpart SF-6D. Disease-specific HRQoL assessment tools were also used but less frequently than generic tools (SGRQ was used by two studies [10, 18], CCQ and PROMIS were used by one each [17, 38]). Likewise, they used different calculation methods and presentation styles even in the studies using similar tool. These issues not only make difficulties in comparison of impact of Covid-19 on HRQoL of patients with Covid-19 between studies, but also confuses policy makers about the use of research findings for policy interventions.

Strengths of the review include- it is a first review of its type to date, as there were no published review on this topic was found. Likewise, studies for this review were searched in multiple databases (PuBMed, Scopus and Medlines), websites of distinguished organisations and reference lists. We used robust selection criteria and the study was reported according PRISMA guidelines. Likewise, we included all eligible studies from all over the world without limiting geographical boundaries. Moreover, we compared the review findings by country, gender, age group, and severity of patients, using wide range of relevant literature published till date.

There are some limitations in this rapid review. First, we included the original articles published in English language only. Second, we included studies published online. There may be other unpublished studies about the impact of Covid-19 on HRQoL of confirmed or suspected patients. Third, we included only those articles which assessed HRQOL of patients with Covid-19 (confirmed or suspected) and excluded other HRQoL studies on general population or with other disease groups, because these were out of our study criteria. Fourth, we also included three papers with clinically suspected Covid-19 patients [23] and both confirmed and suspected patients [17, 35] in this review, as during the start of the Covid-19 pandemic, testing facilities were not widely available and researchers had to include suspected Covid-19 patients as well in their research studies. Therefore, we included these studies in our review according to our inclusion criteria. However, these papers might be slightly impacted the outcomes of the review results. Fifth, we did not assess the quality of the included papers in this review; however, we ensured that the study had required information on important variables of interest (i.e. quality of life scores, types of patients included in the study).

## Conclusions

We concluded that there were higher impact of Covid-19 on HRQoL of Acute Covid (confirmed or suspected) patients compared to Long Covid patients. However the impact was affected by many other factors, such as age, comorbidity, severity of illness of patients and the impact is not reduced considerably as time goes by (i.e. even after two months) [17]. In addition, HRQoL of patients with Covid-19 was considerably lower than the general population during the pandemic. This review also concluded that the impact of Covid-19 on HRQoL of patients from LMICs were considerably lower than the patients from high-income countries (HICs), such as the UK and Norway. Similarly, the HRQoL scores (both physical health and mental health components) were considerably lower among the severe patients who were admitted in ICU compared to moderate patients who were admitted in general wards; the HRQoL scores were found to be lower in female Covid-19 patients, and patients who were in old age (age >60 yrs). Although the long-term impact of Covid-19 is not fully developed, there is a consensus in the included studies that Covid-19 causes long-term problems such as fatigue, cough and shortness of breath, which reduce HRQoL of Covid-19 patients at a considerable level. In addition, most of the factors affecting HRQoL found to be similar for both Acute Covid and Long Covid patients.

There is a need for more studies on Acute Covid (within 4 weeks from the onset of symptoms) and Long Covid after 12 weeks from the onset of symptoms, covering non-hospitalised patients and children and adolescents below 18 years of ag., using standard HRQoL tools (e.g. tools using preference based approach (e.g. EQ-5D, SF-6D) and disease specific tools CCQ, SGRQ) with standard method of calculating HRQoL and presenting estimates (i.e. mean scores for each dimension with SD and 95% CI, median with range, or % of patients facing difficulties for each dimension).

The findings of the review may be useful to the researchers, policy makers, clinicians and those who are interested in the HRQoL of patients affected by Covid-19 pandemic.

## Supporting information

S1 Data Data file related to the paper. (XLSX)
